# A randomized phase II trial of efficacy and safety of the immunotherapy ALECSAT as an adjunct to radiotherapy and temozolomide for newly diagnosed glioblastoma

**DOI:** 10.1093/noajnl/vdab156

**Published:** 2021-10-22

**Authors:** Katja Werlenius, Giuseppe Stragliotto, Michael Strandeus, Malin Blomstrand, Helena Carén, Asgeir S Jakola, Bertil Rydenhag, Dorte Dyregaard, Karine N Dzhandzhugazyan, Alexei F Kirkin, Martin K Raida, Anja Smits, Sara Kinhult

**Affiliations:** 1 Department of Oncology, Sahlgrenska University Hospital, Gothenburg, Sweden; 2 Department of Oncology, Institute of Clinical Sciences, Sahlgrenska Academy, University of Gothenburg, Gothenburg, Sweden; 3 Department of Neurology, Karolinska University Hospital, Stockholm, Sweden; 4 Department of Oncology, Ryhov Hospital, Jönköping, Sweden; 5 Sahlgrenska Center for Cancer Research, Department of Laboratory Medicine, Institute of Biomedicine, Sahlgrenska Academy, University of Gothenburg, Gothenburg, Sweden; 6 Department of Neurosurgery, Sahlgrenska University Hospital, Gothenburg, Sweden; 7 Institute of Neuroscience and Physiology, Department of Clinical Neuroscience, Sahlgrenska Academy, University of Gothenburg, Gothenburg, Sweden; 8 Cytovac A/S, Hørsholm, Denmark; 9 Department of Neuroscience, Neurology, Uppsala University, Uppsala, Sweden; 10 Department of Clinical Sciences, Lund University, Skane University Hospital, Lund, Sweden

**Keywords:** adoptive cell therapy, ALECSAT, glioblastoma, immunotherapy, randomized trial

## Abstract

**Background:**

There is an urgent need for effective treatments against glioblastoma (GBM). In this trial, we investigated the efficacy and safety of an adoptive cell-based immunotherapy.

**Methods:**

Patients with newly diagnosed GBM were recruited at 4 study sites in Sweden. The patients were randomized 1:2 to receive either radiotherapy (RT), 60 Gy/30 fractions, with concomitant and adjuvant temozolomide (TMZ) only, or RT and TMZ with the addition of Autologous Lymphoid Effector Cells Specific Against Tumor (ALECSAT) in an open-label phase II trial. The primary endpoint was investigator-assessed progression-free survival (PFS). The secondary endpoints were survival and safety of ALECSAT.

**Results:**

Sixty-two patients were randomized to either standard of care (SOC) with RT and TMZ alone (n = 22) or SOC with ALECSAT (n = 40). Median age was 57 years (range 38–69), 95% of the patients were in good performance status (WHO 0–1). There was no significant difference between the study arms (SOC vs ALECSAT + SOC) in PFS (7.9 vs 7.8 months; hazard ratio [HR] 1.28; 95% confidence interval [CI] 0.70–2.36; *P* = .42) or in median overall survival (OS) (18.3 vs 19.2 months; HR 1.16, 95% CI 0.58–2.31; *P* = .67). The treatment groups were balanced in terms of serious adverse events (52.4% vs 52.5%), but adverse events ≥grade 3 were more common in the experimental arm (81.0% vs 92.5%).

**Conclusion:**

Addition of ALECSAT immunotherapy to standard treatment with radiochemotherapy was well tolerated but did not improve PFS or OS for patients with newly diagnosed GBM.

**Key Points**

- ALECSAT, an adoptive cell therapy, was added to radiochemotherapy for GBM.
- The treatment did not improve PFS or OS in this randomized phase II trial.
- ALECSAT may be efficacious in other settings, as supported by previous data.

**Importance of the Study**

This study investigated the addition of a specific form of adoptive cell therapy, ALECSAT, to standard of care with RT and TMZ for newly diagnosed GBM. The study did not show any improvement of PFS (primary endpoint) or OS (secondary endpoint) with the addition of ALECSAT. The treatment was well tolerated. Possible factors related to the lack of efficacy are concomitant use of corticosteroids, TMZ-induced lymphopenia, or too low dose of ALECSAT. In the present setting, ALECSAT is not effective as a single immunotherapy modality. Combination strategies may be needed to overcome GBM resistance to immunotherapy.

Glioblastoma (GBM, grade IV astrocytoma) is the most common malignant brain tumor in adults, constituting 48.3% of malignant primary brain tumors.^1^ The worldwide incidence is around 3–4 per 100 000.^2^ Primary treatment for GBM is maximal safe surgical resection followed by radiotherapy (RT) with concomitant and adjuvant temozolomide (TMZ).^3^ Despite multimodal treatment, GBM is associated with a very poor prognosis.^3–5^ In recent years, addition of Tumor-Treating Fields (TTFields, Optune) to adjuvant TMZ has been shown to increase survival.^6^ There is no standard treatment at relapse, but reoperation, alkylating chemotherapy, and re-irradiation can be considered. Thus, there is a large unmet need for new treatments to improve the prognosis for patients with GBM.

Cancer is characterized by the loss of normal cellular regulatory processes, resulting among others in the expression of neoantigens. Bound to human leukocyte antigen (HLA) on the cancer cell surface, these antigens may be recognized by the adaptive immune system, resulting in the activation of antigen-presenting cells (APCs) and cytotoxic T lymphocytes (CTLs) leading to tumor killing.^7^

Adoptive cell therapy (ACT) is a highly personalized immunotherapy that relies on ex vivo activation and expansion of the patients’ own immune cells to recognize and kill the cancer cells when re-introduced to the patient. ACT encompasses tumor-infiltrating lymphocytes, genetically modified T cells expressing novel T-cell receptors, and chimeric antigen receptor T cells.^8–10^

ALECSAT is a form of ACT, developed to induce an immune response against a broad repertoire of cancer–testis antigens (CTAs). Whereas CTAs are silent in normal cells, except germ cells of the testis, they are aberrantly re-expressed in most human solid cancers due to promoter demethylation, rendering them an attractive target for cancer treatment.^11–13^ In the normal situation, T-helper (T_H_) cells and CTLs must recognize the CTAs presented by APCs simultaneously to become activated. When activated, the CTLs target and kill cells that express these specific antigens presented by the APCs. In ALECSAT, the genome of T_H_ cells is demethylated by 5-aza-2′-deoxycytidine treatment. These T_H_ cells can act as both APC and co-stimulatory cells at the same time.^11^ ALECSAT consists of 2 main populations of effector cells: CTLs and natural killer cells.

The ability of ALECSAT to induce a cytotoxic effect in a dose-dependent way has been demonstrated in vitro.^11,14^ ALECSAT has previously been tested in a phase I clinical trial including 25 recurrent GBM patients, a single-arm trial with no comparator. Among the 10 patients who continued ALECSAT after the 3 initial doses, 2 obtained a complete response and 1 patient obtained a partial response.^11^ In addition, ALECSAT has been studied in a randomized phase II trial in recurrent GBM, with bevacizumab and irinotecan as comparators. The results from the interim analysis have been presented, but not published.^15^ ALECSAT did not prolong progression-free survival (PFS) or overall survival (OS), as compared to bevacizumab and irinotecan, and no objective responses to ALECSAT were seen. The treatment appeared well tolerated and no safety concerns were raised. The trial was terminated early, due to the lack of efficacy of ALECSAT in the recurrent setting.

Here we report the results of a randomized phase II trial of the efficacy and safety of ALECSAT as an adjunct to RT and TMZ for patients with newly diagnosed GBM.

## Patients and Methods

### Patients

This open-label, randomized phase II trial enrolled patients, aged 18–70 years, with histologically confirmed newly diagnosed GBM, including gliosarcoma. Patients were recruited at 4 study sites in Sweden (Göteborg, Jönköping, Stockholm, and Lund), from April 1, 2016 until February 22, 2019. Eligible patients were fit for combined RT and TMZ treatment and in World Health Organization (WHO) performance status (PS) 0–2 at the time of inclusion. Prior GBM treatment was an exclusion criterion. Patients were allowed to have systemic steroids, without limitation in dose. Tumor-treating fields treatment and experimental therapies for GBM were not allowed at inclusion or during active treatment within the assigned group. Additional inclusion and exclusion criteria are listed in the synopsis, provided as Supplementary Methods 1.

The trial was conducted in accordance with International Council for Harmonization Good Clinical Practice guidelines. The study protocol and all amendments were approved by the Ethics Committee in Gothenburg (Regional Ethics Review Board, Dnr 1001-15, date of approval January 26, 2016) and by the Swedish Medical Products Agency (EudraCT number 2015-004058-17). All included patients signed written informed consent prior to any study-specific procedure. A review of all collected safety data was performed annually by an independent Data Safety Monitoring Board. The trial was designed by representatives from Cytovac A/S together with the first author and coworkers.

### Treatment

Patients were randomly assigned, in a 1:2 ratio, to receive either standard of care (RT and concomitant and adjuvant TMZ, hereafter called SOC) or SOC plus ALECSAT as an adjunct. Photon RT was delivered, with modern 3D conformal planning technique, at 2 Gy per fraction, to a total dose of 60 Gy, over approximately 6 weeks. During RT, daily TMZ was administered orally, at a dose of 75 mg/m^2^. RT with TMZ had to start within 6 weeks of the diagnostic GBM surgery and was given in accordance with the Swedish national guidelines for high-grade gliomas. Four to five weeks after the completion of RT, all patients were to begin adjuvant TMZ treatment, typically for 6 cycles (TMZ 150-200 mg/m^2^, once daily for 5 days every 28 days).^3^

Patients randomized to SOC + ALECSAT donated blood (300 ml), for the first ALECSAT production, before the start of radiochemotherapy. ALECSAT (at a dose of 1 × 10^7^ to 1 × 10^9^ cells, suspended in 20 ml plasmalyte solution and given intravenously) was administered every 4 weeks for 3 doses, starting at study week 8, approximately 1 week after the completion of RT. Before each ALECSAT dose, the patient donated 200 ml of blood for the subsequent treatment. Following the loading phase, ALECSAT doses were given every 12 weeks starting at week 35. Each ALECSAT preparation was produced by Cytovac A/S (Copenhagen, Denmark) as described previously^11^ and summarized in Supplementary Methods 2. Expression of CT antigens in T_H_ lymphocytes treated with a DNA-demethylating agent was measured in the intermediate step of production. Before release from laboratory and transport to the study sites, the content of the ALECSAT product was analyzed and the in vitro activity was tested against GBM cell line HROG17,^16^ using real-time cytotoxicity assays (Supplementary Methods 2).

**Figure 1.**
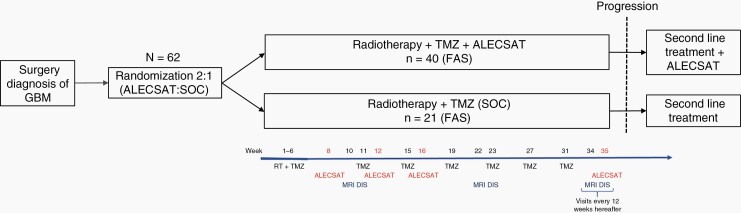
Overview of trial design and study schedule. DIS, disease status evaluation. FAS, full analysis set (or population).

Second-line treatment after disease progression was administered at the discretion of the investigator. Patients were allowed to remain on ALECSAT treatment after disease progression (Figure 1), due to the known risk of pseudoprogression during immunotherapy. Cross-over to treatment with ALECSAT was not allowed for patients randomized to SOC.

### Patient Evaluation and Follow-up

A pathological diagnosis of GBM or gliosarcoma was accepted. IDH1 mutation and *MGMT*-promoter methylation status were analyzed and reported according to local standards. Disease status at baseline was decided using the postoperative MRI, or if not available, the dose planning MRI. Evaluation was performed with MRI and clinical examination every 12 weeks. Patients randomized to the SOC arm followed the same study plan and underwent the same study procedures as the patients in the experimental arm, except for the additional visits for blood donation for ALECSAT production and ALECSAT treatment (meaning 4 extra visits for the ALECSAT patients during the loading phase and 1 extra visit every 3 months during the maintenance phase).

### Outcomes

The primary endpoint was PFS, measured as the time from randomization to the date of investigator-assessed progressive disease (PD) or death by any cause. PD was based on MRI, clinical status, and use of corticosteroids, according to Response Assessment in Neuro-Oncology criteria.^17^ Secondary endpoints were OS, measured from the time of randomization until death for any reason, and the proportion of patients alive at 12 and 24 months after randomization (1-year and 2-year OS). Safety endpoints were adverse events (AEs), safety laboratory parameters, and immune system status.

### Statistical Analysis

Between February 2, 2018 and October 16, 2018, there was a halt in recruitment, during which the study protocol was substantially amended. The primary endpoint was altered from OS to PFS, the secondary endpoint from PFS to OS, and the sample size reduced from 87 to 60, due to economic reasons. The new sample size calculation was based on the assumptions of a median PFS of 6.9 months in the SOC group^3^ and an expected hazard ratio (HR) in favor of the ALECSAT + SOC therapy of 0.54, equivalent to a median PFS of 12.8 months in this group. A one-sided 10% significance level was used to establish the significance of the observed differences. The primary analysis was performed, according to protocol, when 47 events (investigator-assessed progression or death by any cause) had been observed. Data-base lock (DBL) for this analysis was on November 14, 2019, but patients continued treatment according to protocol until the end of study (EoS, February 24, 2020). The data were signed by the investigators after EoS. Here we present the final analysis at DBL on the signed and locked dataset. Statistical analysis was performed using SAS 9.4 PROC PHREG and LIFE TEST. Descriptive statistics were summarized using counts and proportions, means were presented with standard deviations, and medians with range. For comparison between groups, Fisher’s exact test (lowest 1-sided *P* value multiplied by 2) was used for dichotomous variables, and the Mann–Whitney *U* test was used for continuous variables. The PFS and OS were analyzed using Kaplan–Meier survival estimates. Comparisons between treatment groups were analyzed by log-rank test.

## Results

### Patient Characteristics

**Figure 2.**
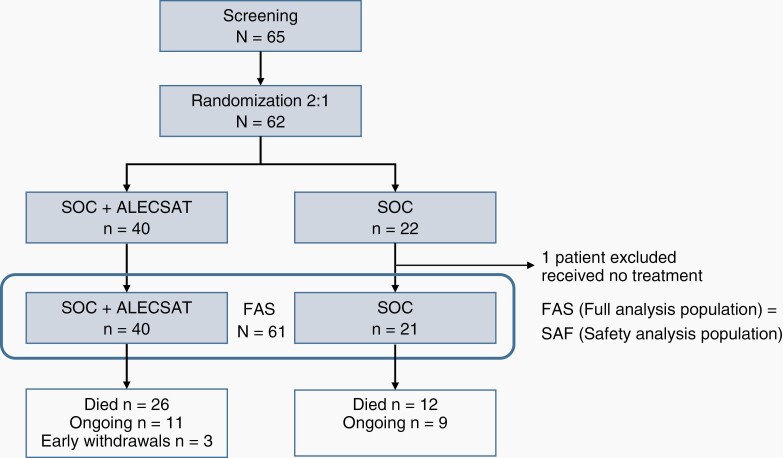
Randomization and study populations.

Sixty-five patients were screened and 62 patients were randomized in the trial, 22 in the SOC group and 40 in the SOC + ALECSAT group. One patient in the SOC group did not receive oncological treatment at all, due to a serious fungal infection, unrelated to tumor treatment, and was excluded from the full analysis set (FAS). The FAS includes all 61 patients who entered the trial and received at least one trial treatment. The randomization and study arms are presented in Figure 2. Three patients in the experimental arm, still alive at DBL, had terminated the trial by early withdrawal, due to PD with inability to continue to comply with trial procedures (n = 2) or PD and that the blood donation could put the patient at risk (n = 1).

The treatment groups were well balanced in terms of sex and PS, with a majority of men (n = 36, 59%) and most patients (n = 58, 95%) were in PS WHO 0 or 1 at baseline. The median age of the SOC group was higher, 62 years (range 44–69), compared to 55.5 years (range 38–69) in the SOC + ALECSAT group. Complete resection was achieved in 47.6 % in SOC arm and 42.5 % in SOC + ALECSAT. One single patient in the SOC + ALECSAT group had a multifocal disease, all other patients had single tumors only. All tumors, but one, were IDH wildtype (IDHwt). There were significantly more patients with unmethylated *MGMT* status in the SOC + ALECSAT group (n = 28; 70%) as compared to the SOC group (n = 7; 33%). Relatively more patients in the SOC group used corticosteroids at baseline (n = 10, 48%), compared to the SOC + ALECSAT group (n = 10, 25%; Table 1).

**Table 1.** Baseline Characteristics (FAS)

Variable	SOC (n = 21) n (%)	SOC + ALECSAT (n = 40) n (%)	Total (N = 61) N (%)
Age			
Median, years (range)	62.0 (44–69)	55.5 (38–69)	57.0 (38–69)
Mean, years (SD)	59.9 (±7.3)	55.2 (±9.0)*	56.8 (± 8.6)
Gender			
Female	9 (42.9)	16 (40.0)	25 (41.0)
Male	12 (57.1)	24 (60.0)	36 (59.0)
Performance status^a^			
0	10 (47.6)	19 (47.5)	29 (47.5)
1	10 (47.6)	19 (47.5)	29 (47.5)
2	1 (4.8)	2 (5.0)	3 (4.9)
Type of surgery			
Complete resection	10 (47.6)	17 (42.5)	27 (44.3)
Partial resection	8 (38.1)	21 (52.5)	29 (47.5)
Biopsy	3 (14.3)	2 (5.0)	5 (8.2)
Tumor location^b^			
Frontal	5 (23.8)	13 (32.5)	18 (29.5)
Temporal	11 (52.4)	17 (42.5)	28 (45.9)
Parietal	5 (23.8)	7 (17.5)	12 (19.7)
Occipital	1 (4.8)	6 (15.0)	7 (11.5)
Multifocal^c^	0 (0)	1 (2.5)	1 (1.6)
Tumor characteristics			
IDH1wt	21 (100)	39 (97.5)	60 (98.4)
IDH1mut	0 (0)	1 (2.5)	1 (1.6)
MGMT status			
Unmethylated	7 (33.3)	28 (70.0)	35 (57.4)
Methylated	14 (66.7)	12 (30.0)**	26 (42.6)
Use of steroids at baseline			
Yes	10 (47.6)	10 (25.0)	20 (32.8)
No	11 (52.4)	30 (75.0)	41 (67.2)
Median time from surgery to start of oncological treatment^d^ (days and range)	35 (23–43)	31 (22–42)	32 (22–43)

^a^Performance status, according to Eastern Cooperative Oncology Group/WHO.

^b^Five patients had unifocal tumors engaging more than 1 lobe.

^c^Multifocal tumor, defined as at least 2 separate contrast-enhancing lesions.

^d^Waiting time from surgery to start of radiotherapy and TMZ.

*P = .046, **P = .013.

### Treatment Characteristics

The median time from surgery to the start of RT and TMZ was 32 days (Table 1). RT was given as planned to 97% of the patients, with 2 Gy per fraction to a median dose of 60 Gy in both treatment arms (Table 2). One patient in each group stopped RT earlier than planned, due to epileptic seizures requiring hospitalization and infection, respectively. The treatment groups were well balanced in terms of concomitant TMZ treatment, with a median duration of 43 days (range 24–48 days). There were no dose reductions during treatment, but 6 patients (15%) in the SOC + ALECSAT group and 3 patients (14.3%) in the SOC group stopped concomitant TMZ treatment earlier than planned (Table 2). The most common reasons for terminating concomitant TMZ early were thrombocytopenia (n = 5), followed by rashes (n = 2), infection, and liver toxicity (one of each).

**Table 2.** Treatment Characteristics (FAS)

Variable	SOC (n = 21)	SOC + ALECSAT (n = 40)
*Radiotherapy*		
Total dose, Gy (median and range)	60.0 (58.0–60.0)	60.0 (38.0–60.0)
Number of fractions, n (range)	30 (29–30)	30 (19–30)
Treatment given as planned, n (%)	20 (95.2%)	39 (97.5%)
*Concomitant TMZ*		
Daily dose in mg, median (range)	140 (120–160)	140 (120–180)
Duration of TMZ treatment, days (median and range)	43 (24–48)	43 (28–48)
Treatment given as planned, n (%)	18 (85.7)	34 (85.0)
*Adjuvant TMZ*		
Number of cycles, mean (SD)	4.2 (±2.5)	4.4 (±2.0)
Median (range)	6.0 (0–8)	5.0 (0–9)
Patients completed 6 cycles, n (%)	12 (57.1)	18 (45.0)
*ALECSAT*		
Loading phase		
At least 2 doses, n (%)	–	40 (100)
Completed 3 loading doses, n (%)	–	35 (87.5)
Maintenance phase		
At least one dose, n (%)	–	28 (70.0)
Received 4 maintenance doses, n (%)	–	12 (30.0)
Received 7 maintenance doses, n (%)	–	6 (15.0)
Continued ALECSAT beyond PD, n (%)	–	25/34 (73.5)
*Second-line treatment after PD*		
Received any second-line treatment	12/15 (80.0)	27/34 (79.4)

A total of 5 patients did not start adjuvant TMZ treatment, due to bone marrow toxicity (n = 3) or suspected disease progression (n = 2). Patients in the SOC group received a median of 6 adjuvant TMZ cycles (range 0–7) versus 5 cycles (range 0–9) in the SOC + ALECSAT group. More patients (57%) in the SOC group, versus the SOC + ALECSAT group (45%) completed the planned 6 cycles of adjuvant TMZ (Table 2). The main reason for not completing all adjuvant TMZ cycles was disease progression.

All patients randomized to the experimental arm received at least 2 loading doses of ALECSAT. Two patients missed ALECSAT treatments (1 and 2 doses, respectively) due to technical manufacturing problems. The number of patients receiving ALECSAT dropped markedly from the loading phase to the maintenance phase with only 28 subjects (70%) receiving the first maintenance dose (Table 2). A total of 8 patients (20%) had to stop maintenance treatment with ALECSAT earlier than planned due to the closure of the study.

As mentioned above, 48% of patients in the SOC group used corticosteroids at baseline, compared to 25% in the experimental arm. At the end of RT, the proportion of patients on steroids had decreased to 38% in the SOC group, but increased to 60% in the SOC + ALECAT group. The trend over time of more patients in the experimental arm using steroids remained. A clear difference in the use of corticosteroids between study sites was noted.

After progression, 39 patients received second-line treatment, the most common being single Lomustine. Seventy-four percent (25 of 34) of patients continued ALECSAT treatment after progression (Table 2).

### ALECSAT Product Characteristics

The ALECSAT products contained a median of 117 million cells at the first loading dose (week 8). At the following dose (week 12), the cell amount in the ALECSAT products had decreased to a median of 38.5 million. Hereafter, the cell number per treatment remained relatively stable, at lower levels than initially, for most patients (Supplementary Table 1). In addition to the reduction in the total number of cells, the levels of several subpopulations of T lymphocytes also decreased starting from the second treatment (Supplementary Tables 1–3). These reductions appeared to coincide with the observed decrease, within normal range, in peripheral leukocytes, lymphocytes, neutrophils, CD4+ cells, and platelets. Similar decreases in blood counts were seen in the SOC group (data not shown).

The cell viability, at the release from the laboratory, was stable with a median viability of around 95% over the course of the trial (Supplementary Table 1). Information on the lymphocyte subpopulations in the final product and expression of CTA in T_H_ cells treated with 5-aza-2′-deoxycytidine is given in Supplementary Tables 1–4.

There was a clear and stable dose response in the cytotoxicity test of ALECSAT cells in vitro throughout the trial (Supplementary Table 5).

### Survival

**Figure 3.**
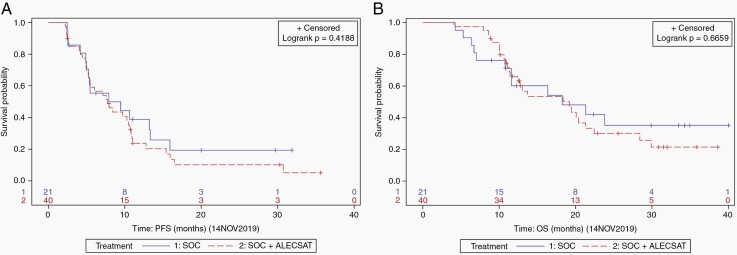
(A) Progression-free survival and (B) overall survival.

The PFS was 7.9 months in the SOC group compared to 7.8 months in the SOC + ALECSAT group (Figure 3A). The difference between the treatment groups was not statistically significant (HR 1.28; 95% confidence interval [CI] 0.70–2.36; *P* = .42). The median OS was 18.3 months in the SOC group and 19.2 months in the SOC + ALECSAT group (Figure 3B). The difference was not statistically significant (HR 1.16; 95% CI 0.58–2.31; *P* = .67).

The 1-year probability of survival was 60.2% (95% CI 38.5–81.8) in the SOC group and 66.1% (95% CI 51.1–81.2) in the experimental arm. The 2-year probability of survival was 35.1% (95% CI 12.3–57.9) in the SOC group, compared to 30.0% (95% CI 14.1–46.0) in the SOC + ALECSAT group. The differences between treatment groups were not statistically significant.

### Toxicity and Safety

All patients in the trial reported AEs related to SOC and more than half (57.5%) of the patients in the SOC + ALECSAT group experienced AEs possibly related to ALECSAT, as assessed by the investigator. AEs of ≥CTCAE grade 3 were more common in the experimental group than in the SOC group (92.5% vs 81.0%), but the treatment arms were well balanced in terms of patients with serious adverse events (SAEs), 52.5% versus 52.4%. There was one fatal SAE in the SOC group, as one patient died in a gastrointestinal hemorrhage. This particular patient terminated concomitant TMZ treatment early due to thrombocytopenia, but the platelet count was normalized 2 months before the event, and no adjuvant TMZ treatment was given according to the patient’s wish. The patient had a relatively high dose of steroids at the time of death (4 mg betamethasone daily). Reported AEs, somewhat more commonly reported in the SOC + ALECSAT group, were brain edema, mild headache, and infections (Table 3). An SAE of retinal infiltrates in a patient with diabetes and visual disturbance in the medical history was reported 3 months after the last ALECSAT dose. This event was considered possibly related to ALECSAT and TMZ treatment, with an alternative explanation according to the investigator being a concurrent illness. Two patients developed spinal metastasis (1 patient in each treatment arm) and 1 patient in the SOC + ALECSAT group had distant metastases to the right lung, diaphragm, and both kidneys at autopsy. These 3 patients all had IDHwt GBMs, 2 with unmethylated and 1 with methylated *MGMT* status.

**Table 3.** Summary of Adverse Events

	SOC (n = 21)		SOC + ALECSAT (n = 40)	
	AEs	Patients with AEs	AEs	Patients with AEs
	n	n (%)	n	n (%)
Any AE	445	21 (100)	1042	40 (100)
AE ≥grade 3	76	17 (81.0)	152	37 (92.5)
AEs related to ALECSAT	0	0 (0.0)	201	23 (57.5)
AEs related to SOC	213	21 (100)	477	40 (100)
AEs leading to discontinued treatment	35	11 (52.4)	33	22 (55.0)
Any SAE*	19	11 (52.4)	40	21 (52.5)
SAEs related to ALECSAT	0	0 (0.0)	12	9 (22.5)
SAEs related to SOC	7	4 (19.0)	11	8 (20.0)
SAEs leading to death*	1	1 (4.8)	0	0 (0.0)
AEs of special interest**				
Brain edema	4	3 (14.3)	11	10 (25.0)
Fatigue	33	19 (90.5)	77	38 (95.0)
Headache	20	7 (33.3)	56	30 (75.0)
Seizures	19	8 (38.1)	43	17 (42.5)
Infections	22	12 (57.1)	59	28 (70.0)
Thromboembolism	7	5 (23.8)	20	12 (30.0)
Leukopenia ≥grade 3	12	6 (28.6)	9	9 (22.5)
Thrombocytopenia ≥grade 3	5	4 (19.0)	4	4 (10.0)

*Disease progressions not included.

**Any grade if not otherwise stated.

## Discussion

Here we report the results of a phase II trial on the efficacy and safety of ALECSAT as an add-on therapy to RT and TMZ in patients with newly diagnosed GBM. The trial did not meet the primary endpoint of improved PFS with the addition of ALECSAT to SOC treatment (7.8 vs 7.9 months) as compared to SOC only. The OS was comparable between the treatment groups (19.2 vs 18.3 months), and these small differences were not statistically significant. In the SOC + ALECSAT group, patients were significantly younger, but it was also more common with unmethylated *MGMT* promoter. Adjusting for these unbalances at baseline did not yield any significant survival differences between the study groups. The PFS and OS of both treatment arms in our study are comparable with outcome reported from randomized phase III trials,^5,18,19^ as well as other immunotherapy trials in newly diagnosed GBM.^20–22^

Our results are not in accordance with the earlier reported efficacy of ALECSAT as a single agent, observed in a phase I clinical trial (NCT01588769), where 3 out of 25 patients with recurrent GBM obtained a treatment response.^11^ Several factors need to be considered trying to explain the lack of efficacy of ALECSAT in the present study setting. In the phase I study, the patients did not receive chemotherapy during ALECSAT treatment. It cannot be excluded that the combination with TMZ in the present trial had a negative impact on the efficacy of ALECSAT. In fact, the median number of cells in the ALECSAT product was reduced by ≥50% after the initiation of TMZ treatment. Since batches were manufactured with the same number of cells initially, this suggests that TMZ may have affected the ability of T cells to proliferate during the manufacturing of ALECSAT.

Furthermore, in the phase 1 trial of recurrent GBM, the average lymphocyte cell number in ALECSAT preparation was 156 million. In the present study, on average, less than 100 million cells were present in the ALECSAT product. It cannot be ruled out that this contributed to a negative clinical impact. Indeed, earlier studies demonstrated a positive correlation between the number of ALECSAT cells and the number of killed cancer cells in vitro, stressing the importance of the number of infused cells.^11,14^ A clear dose response of cytotoxicity of the ALECSAT products in vitro was also seen in the present study.

Previous studies in patients with newly diagnosed GBM have shown significant and long-lasting lymphopenia, with severe reductions of CD4 counts, following treatment with RT and TMZ.^23,24^ Corticosteroids, commonly used to treat peritumoral edema in GBM patients, have well-known negative effects on lymphocyte function and increase immune suppression in the tumor microenvironment.^25,26^ Increased use of corticosteroids during chemoradiotherapy has shown to be an independent risk factor for developing acute severe lymphopenia.^27,28^ There is also increasing evidence that corticosteroids might negatively impact survival in GBM and may hamper the efficacy of immunotherapies.^25,29^ Considering this, it would have been desirable with no or very low doses of steroids during ALECSAT treatment. In fact, the proportion of patients using corticosteroids in the experimental arm was only 25% at baseline. However, this proportion increased to 60% at the end of RT, before the start of ALECSAT treatment, demonstrating the difficulties in anticipating future steroid need at the time of inclusion. We observed significant decreases in peripheral lymphocytes, CD4+ and CD56+ cells following chemoradiotherapy in both treatment arms, as compared to baseline. This cell reduction may have counteracted the ACT.

In our preclinical study, testing ALECSAT on autologous GBM-derived cancer stem cells, the in vitro effect correlated significantly with the blood count of CD4+ cells in the patient.^14^ This suggests that there may be a benefit in collecting cells for ALECSAT preparation at an even earlier stage, when patients generally have higher blood counts, as also seen in our trial. An increase in the proportion of stem cell memory cells and central memory cells in ACT has been suggested to improve the persistence and antitumor efficacy.^30^ A new version of ALECSAT (ALECSAT-2) has recently been developed that permits generation of a larger number of the effector cells with higher expression of CD62L. ALECSAT-2 is currently being tested in a clinical trial with triple-negative breast cancer patients (NCT04609215).

The reported AEs in our study were consistent with known side effects from RT and TMZ or symptoms present at disease progression of GBM. During the study, brain edema was reported as SAE, with possible but not evident relation to ALECSAT, for 4 patients in the experimental arm. Even though the frequency of AEs was somewhat higher in the SOC + ALECSAT group, the distribution of AEs was comparable between treatment arms. SAEs were reported with similar frequencies in both treatment groups, and no new safety signals were observed in the trial.

Data from early immunotherapy studies have indicated a potentially important role for immunotherapy in the future treatment of GBM,^11,31–33^ but at the same time, several clinical trials in recent years have failed to demonstrate efficacy.^20,34,35^ As pointed out in recent reviews, there are several obstacles underlying the immunotherapy resistance of GBM that need to be overcome: the low immunogenicity of the tumor itself, the immune privilege of the CNS, and the immune-suppressive microenvironment. A combination of different immunotherapy strategies might be needed to overcome these impediments.^36,37^

### Limitations

There are obvious limitations of our study. PFS (chosen due to economical restrictions faced by the sponsor) is not the best choice of primary endpoint in GBM, due to the well-known risk of pseudoprogression after RT with TMZ.^17^ In this trial, however, the mature OS data were in concordance with PFS, and therefore, the choice of PFS is unlikely to have biased the study result. Furthermore, the trial was open-label, with an inherent risk of bias among investigators in the reporting of AEs and PD. However, this possible bias would unlikely have affected OS, and it was considered ethically unacceptable for patients to donate blood without receiving a potential positive treatment effect. Patient numbers were based on a sample size calculation hypothesizing a large positive treatment effect of ALECSAT (HR 0.54), which was very ambitious, but considered reasonable for such a complex and costly treatment like ALECSAT. The complete lack of efficacy signal in OS data in this study, however, shows that ALECSAT was not effective in the present setting.

## Conclusion

The addition of ALECSAT immunotherapy to RT and TMZ did not improve PFS or OS for patients with newly diagnosed GBM. The combination with TMZ and corticosteroids may have had a negative impact on the efficacy of ALECSAT treatment. The treatment was well tolerated and future studies should consider a different approach.

## Supplementary Material

vdab156_suppl_Supplementary_Tables

vdab156_suppl_Supplementary_Data_S1

vdab156_suppl_Supplementary_Data_S2
