# Supplementary material for: A randomized phase II trial of efficacy and safety of the immunotherapy ALECSAT as an adjunct to radiotherapy and temozolomide for newly diagnosed glioblastoma
Source: Neurooncol Adv. 2021 Oct 22;3(1):vdab156. doi: 10.1093/noajnl/vdab156 (PMC8577524; doi:10.1093/noajnl/vdab156)
Supplement: vdab156_suppl_Supplementary_Tables [file vdab156_suppl_supplementary_tables.docx]

**Supplementary tables: ALECSAT product characteristics**

**S1. ALECSAT: Total number of cells, viability and number of CD4+ cells by visit (FAS)**

|  | | Total number of cells (million) (N=40) | Viability (%) (N=40) | CD4+ cells (%) (N=40) | Number of CD4+ cells (million) (N=40) |
| --- | --- | --- | --- | --- | --- |
|  | |  |  |  |  |
|  |  |  |  |  |  |
|  |  |  |  |  |  |
| Week8 ALECSAT patients | n | 39 | 39 | 39 | 39 |
|  | Mean (SD) | 115.26 (63.31) | 0.96 ( 0.02) | 0.15 (0.11) | 18.83 (23.13) |
|  | Standard error of the mean | 10.14 | 0.00 | 0.02 | 3.70 |
|  | Median | 117.00 | 0.96 | 0.14 | 14.30 |
|  | 95 % CI lower - upper | 94.73 - 135.78 | 0.95 - 0.96 | 0.11 - 0.18 | 11.33 - 26.32 |
|  | Min - Max | 9.00 - 261.00 | 0.91 - 0.99 | 0.00 - 0.54 | 0.00 - 137.70 |
|  | Missing | 1 (2.50) | 1 (2.50) | 1 (2.50) | 1 (2.50) |
|  |  |  |  |  |  |
| Week12 ALECSAT patients | n | 38 | 38 | 38 | 38 |
|  | Mean (SD) | 49.50 (37.05) | 0.95 (0.03) | 0.22 (0.18) | 9.30 (7.29) |
|  | Standard error of the mean | 6.01 | 0.00 | 0.03 | 1.18 |
|  | Median | 38.50 | 0.96 | 0.18 | 7.11 |
|  | 95 % CI lower - upper | 37.32 - 61.68 | 0.94 - 0.96 | 0.16 - 0.28 | 6.90 - 11.69 |
|  | Min - Max | 10.00 - 149.00 | 0.90 - 1.00 | 0.02 - 0.89 | 0.65 - 27.52 |
|  | Missing | 2 (5.00) | 2 (5.00) | 2 (5.00) | 2 (5.00) |
|  |  |  |  |  |  |
| Week16 ALECSAT patients | n | 37 | 37 | 37 | 37 |
|  | Mean (SD) | 59.38 (39.39) | 0.96 (0.02) | 0.22 (0.15) | 12.43 (10.62) |
|  | Standard error of the mean | 6.48 | 0.00 | 0.03 | 1.75 |
|  | Median | 45.00 | 0.96 | 0.17 | 9.20 |
|  | 95 % CI lower - upper | 46.25 - 72.51 | 0.95 - 0.96 | 0.17 - 0.27 | 8.89 - 15.97 |
|  | Min - Max | 6.00 - 161.00 | 0.92 - 1.00 | 0.03 - 0.62 | 0.66 - 40.25 |
|  | Missing | 3 (7.50) | 3 (7.50) | 3 (7.50) | 3 (7.50) |
| Week34/35AB | n | 28 | 28 | 28 | 28 |
|  | Mean (SD) | 66.09 (39.55) | 0.95 (0.03) | 0.29 (0.23) | 14.79 (11.28) |
|  | Standard error of the mean | 7.47 | 0.00 | 0.04 | 2.13 |
|  | Median | 59.75 | 0.96 | 0.24 | 11.03 |
|  | 95 % CI lower - upper | 50.75 - 81.43 | 0.94 - 0.96 | 0.20 - 0.37 | 10.42 - 19.17 |
|  | Min - Max | 16.00 - 161.00 | 0.88 - 0.99 | 0.05 - 0.86 | 1.95 - 56.68 |
|  | Missing | 12 (30.00) | 12 (30.00) | 12 (30.00) | 12 (30.00) |

**S2. ALECSAT: Number of CD3+CD8+, CD8+CD62L+ and CD8+CD27+ cells by visit (FAS)**

|  | | CD3+CD8+ cells (%) (N=40) | Number of CD3+CD8+ cells (million) (N=40) | CD8+CD62L+ cells  (% of CD3+CD8+ cells) (N=40) | CD8+CD27+ cells (%) (N=40) | Number of CD8+CD27+ cells (million) (N=40) |
| --- | --- | --- | --- | --- | --- | --- |
|  |  |  |  |  |  |  |
|  |  |  |  |  |  |  |
| Week8 ALECSAT patients | n | 39 | 39 | 39 | 39 | 39 |
|  | Mean (SD) | 0.43 (0.23) | 47.76 (36.00) | 0.68 (0.20) | 0.15 (0.11) | 16.73 16.05) |
|  | Standard error of the mean | 0.04 | 5.76 | 0.03 | 0.02 | 2.57 |
|  | Median | 0.40 | 38.88 | 0.73 | 0.12 | 12.60 |
|  | 95 % CI lower - upper | 0.35 - 0.50 | 36.09 - 59.43 | 0.62 - 0.74 | 0.11 - 0.18 | 11.53 - 21.93 |
|  | Min - Max | 0.09 - 0.96 | 2.97 - 150.45 | 0.26 - 0.92 | 0.02 - 0.44 | 0.66 - 63.75 |
|  | Missing | 1 (2.50) | 1 (2.50) | 1 (2.50) | 1 (2.50) | 1 (2.50) |
|  |  |  |  |  |  |  |
| Week12 ALECSAT patients | n | 38 | 38 | 38 | 38 | 38 |
|  | Mean (SD) | 0.53 (0.24) | 26.03 (21.78) | 0.70 (0.16) | 0.18 (0.17) | 8.31 (8.95) |
|  | Standard error of the mean | 0.04 | 3.53 | 0.03 | 0.03 | 1.45 |
|  | Median | 0.55 | 22.71 | 0.73 | 0.12 | 5.10 |
|  | 95 % CI lower - upper | 0.45 - 0.61 | 18.87 - 33.19 | 0.65 - 0.75 | 0.12 - 0.23 | 5.36 - 11.25 |
|  | Min - Max | 0.10 - 0.92 | 1.95 - 91.14 | 0.31 - 0.95 | 0.00 - 0.62 | 0.00 - 32.68 |
|  | Missing | 2 (5.00) | 2 (5.00) | 2 (5.00) | 2 (5.00) | 2 (5.00) |
|  |  |  |  |  |  |  |
| Week16 ALECSAT patients | n | 37 | 37 | 37 | 37 | 37 |
|  | Mean (SD) | 0.60 (0.17) | 35.76 (28.12) | 0.74 (0.16) | 0.25 (0.17) | 14.64 (15.20) |
|  | Standard error of the mean | 0.03 | 4.62 | 0.03 | 0.03 | 2.50 |
|  | Median | 0.60 | 24.85 | 0.79 | 0.21 | 9.12 |
|  | 95 % CI lower - upper | 0.54 - 0.65 | 26.39 - 45.14 | 0.69 - 0.80 | 0.20 - 0.31 | 9.57 - 19.71 |
|  | Min - Max | 0.26 - 0.90 | 4.32 - 122.98 | 0.32 - 0.95 | 0.04 - 0.68 | 1.26 - 68.64 |
|  | Missing | 3 (7.50) | 3 (7.50) | 3 (7.50) | 3 (7.50) | 3 (7.50) |
| Week34/35AB | n | 28 | 28 | 28 | 28 | 28 |
|  | Mean (SD) | 0.61 (0.19) | 38.49 (21.90) | 0.76 (0.16) | 0.23 (0.14) | 13.78 (7.91) |
|  | Standard error of the mean | 0.04 | 4.14 | 0.03 | 0.03 | 1.49 |
|  | Median | 0.61 | 33.60 | 0.78 | 0.21 | 12.85 |
|  | 95 % CI lower - upper | 0.54 - 0.68 | 30.00 - 46.98 | 0.69 - 0.82 | 0.18 - 0.29 | 10.71 - 16.85 |
|  | Min - Max | 0.30 - 0.90 | 4.80 - 94.55 | 0.35 - 0.97 | 0.03 - 0.64 | 0.48 - 32.64 |
|  | Missing | 12 (30.00) | 12 (30.00) | 12 (30.00) | 12 (30.00) | 12 (30.00) |

**S3. ALECSAT: Number of CD3-CD56+, CD56+CD62L+ and CD3-CD56- cells by visit (FAS)**

|  | | CD3-CD56+ cells (%) (N=40) | Number of CD3-CD56+ cells (million) (N=40) | CD56+CD62L+ cells  (% of CD3-CD56+ cells) (N=40) | CD3-CD56- cells (%) (N=40) | Number of CD3-CD56- cells (million) (N=40) |
| --- | --- | --- | --- | --- | --- | --- |
|  |  |  |  |  |  |  |
|  |  |  |  |  |  |  |
| Week8 ALECSAT patients | n | 39 | 39 | 39 | 39 | 39 |
|  | Mean (SD) | 0.33 (0.22) | 40.62 (39.50) | 0.47 (0.18) | 0.10 (0.07) | 10.58 (8.15) |
|  | Standard error of the mean | 0.04 | 6.32 | 0.03 | 0.01 | 1.31 |
|  | Median | 0.27 | 37.26 | 0.44 | 0.09 | 8.60 |
|  | 95 % CI lower - upper | 0.26 - 0.40 | 27.82 - 53.42 | 0.41 - 0.53 | 0.07 - 0.12 | 7.93 - 13.22 |
|  | Min - Max | 0.01 - 0.73 | 0.86 - 185.31 | 0.04 - 0.83 | 0.01 - 0.28 | 0.27 - 32.48 |
|  | Missing | 1 (2.50) | 1 (2.50) | 1 (2.50) | 1 (2.50) | 1 (2.50) |
|  |  |  |  |  |  |  |
| Week12 ALECSAT patients | n | 38 | 38 | 37 | 38 | 38 |
|  | Mean (SD) | 0.22 (0.20) | 11.79 (15.85) | 0.43 (0.16) | 0.10 (0.08) | 4.96 (4.70) |
|  | Standard error of the mean | 0.03 | 2.57 | 0.03 | 0.01 | 0.76 |
|  | Median | 0.14 | 4.05 | 0.43 | 0.09 | 2.89 |
|  | 95 % CI lower - upper | 0.16 - 0.28 | 6.58 - 17.00 | 0.38 - 0.48 | 0.08 - 0.13 | 3.41 - 6.51 |
|  | Min - Max | 0.00 - 0.72 | 0.00 - 67.05 | 0.13 - 0.74 | 0.01 - 0.29 | 0.13 - 17.86 |
|  | Missing | 2 (5.00) | 2 (5.00) | 3 (7.50) | 2 (5.00) | 2 (5.00) |
|  |  |  |  |  |  |  |
| Week16 ALECSAT patients | n | 37 | 37 | 37 | 37 | 37 |
|  | Mean (SD) | 0.18 (0.14) | 11.19 (13.07) | 0.46 (0.23) | 0.07 (0.06) | 3.77 (3.65) |
|  | Standard error of the mean | 0.02 | 2.15 | 0.04 | 0.01 | 0.60 |
|  | Median | 0.15 | 7.15 | 0.46 | 0.06 | 2.16 |
|  | 95 % CI lower - upper | 0.13 - 0.22 | 6.83 - 15.55 | 0.38 - 0.53 | 0.05 - 0.09 | 2.56 - 4.99 |
|  | Min - Max | 0.00 - 0.52 | 0.00 - 67.62 | 0.00 - 0.86 | 0.01 - 0.20 | 0.31 - 16.15 |
|  | Missing | 3 (7.50) | 3 (7.50) | 3 (7.50) | 3 (7.50) | 3 (7.50) |
| Week34/35AB | n | 28 | 28 | 28 | 28 | 28 |
|  | Mean (SD) | 0.17 (0.14) | 13.73 (18.72) | 0.54 (0.22) | 0.07 (0.04) | 4.68 (4.82) |
|  | Standard error of the mean | 0.03 | 3.54 | 0.04 | 0.01 | 0.91 |
|  | Median | 0.15 | 7.48 | 0.52 | 0.07 | 3.50 |
|  | 95 % CI lower - upper | 0.11 - 0.22 | 6.47 - 20.99 | 0.46 - 0.63 | 0.05 - 0.08 | 2.81 - 6.55 |
|  | Min - Max | 0.01 - 0.54 | 0.32 - 82.08 | 0.10 - 1.00 | 0.01 - 0.16 | 0.51 - 24.15 |
|  | Missing | 12 (30.00) | 12 (30.00) | 12 (30.00) | 12 (30.00) | 12 (30.00) |

**S4: ALECSAT: Melanoma antigen expression by visit (FAS)**

|  | | MAGE-A1 (N=40) | MAGE-A3 (N=40) | MAGE-A4 (N=40) | MAGE-A6 (N=40) | MAGE-A10 (N=40) | MAGE-A12 (N=40) |
| --- | --- | --- | --- | --- | --- | --- | --- |
|  |  |  |  |  |  |  |  |
|  |  |  |  |  |  |  |  |
| Week8 ALECSAT patients | n | 33 | 33 | 33 | 33 | 33 | 33 |
|  | Mean (SD) | 0.68 (1.04) | 0.75 (0.48) | 0.33 (0.27) | 0.35 (0.26) | 0.02 (0.01) | 0.17 (0.14) |
|  | Standard error of the mean | 0.18 | 0.08 | 0.05 | 0.05 | 0.00 | 0.02 |
|  | Median | 0.36 | 0.66 | 0.26 | 0.27 | 0.01 | 0.13 |
|  | 95 % CI lower - upper | 0.31 - 1.05 | 0.58 - 0.92 | 0.23 - 0.42 | 0.25 - 0.44 | 0.01 - 0.02 | 0.12 - 0.21 |
|  | Min - Max | 0.10 - 4.98 | 0.05 - 2.54 | 0.04 - 1.33 | 0.06 - 1.48 | 0.00 - 0.05 | 0.02 - 0.73 |
|  | Missing | 7 (17.50) | 7 (17.50) | 7 (17.50) | 7 (17.50) | 7 (17.50) | 7 (17.50) |
|  |  |  |  |  |  |  |  |
| Week12 ALECSAT patients | n | 26 | 26 | 26 | 26 | 26 | 26 |
|  | Mean (SD) | 0.34 (0.19) | 0.64 (0.48) | 0.28 (0.16) | 0.26 (0.20) | 0.02 (0.02) | 0.13 (0.10) |
|  | Standard error of the mean | 0.04 | 0.09 | 0.03 | 0.04 | 0.00 | 0.02 |
|  | Median | 0.27 | 0.53 | 0.27 | 0.20 | 0.01 | 0.11 |
|  | 95 % CI lower - upper | 0.26 - 0.41 | 0.44 - 0.83 | 0.21 - 0.35 | 0.18 - 0.34 | 0.01 - 0.03 | 0.09 - 0.16 |
|  | Min - Max | 0.09 - 0.74 | 0.15 - 2.44 | 0.05 - 0.78 | 0.00 - 0.81 | 0.00 - 0.08 | 0.03 - 0.47 |
|  | Missing | 14 (35.00) | 14 (35.00) | 14 (35.00) | 14 (35.00) | 14 (35.00) | 14 (35.00) |
|  |  |  |  |  |  |  |  |
| Week16 ALECSAT patients | n | 31 | 31 | 31 | 31 | 31 | 31 |
|  | Mean (SD) | 0.26 (0.17) | 0.49 (0.32) | 0.27 (0.23) | 0.19 (0.10) | 0.01 (0.02) | 0.09 (0.06) |
|  | Standard error of the mean | 0.03 | 0.06 | 0.04 | 0.02 | 0.00 | 0.01 |
|  | Median | 0.22 | 0.35 | 0.19 | 0.17 | 0.01 | 0.08 |
|  | 95 % CI lower - upper | 0.20 - 0.32 | 0.37 - 0.61 | 0.19 - 0.35 | 0.16 - 0.23 | 0.01 - 0.02 | 0.07 - 0.11 |
|  | Min - Max | 0.07 - 1.00 | 0.12 - 1.63 | 0.06 - 1.22 | 0.05 - 0.42 | 0.00 - 0.09 | 0.02 - 0.26 |
|  | Missing | 9 (22.50) | 9 (22.50) | 9 (22.50) | 9 (22.50) | 9 (22.50) | 9 (22.50) |
|  |  |  |  |  |  |  |  |
| Week34/35AB | n | 25 | 25 | 25 | 25 | 25 | 25 |
|  | Mean (SD) | 0.23 (0.12) | 0.51 (0.30) | 0.28 (0.16) | 0.20 (0.12) | 0.01 (0.01) | 0.11 (0.06) |
|  | Standard error of the mean | 0.02 | 0.06 | 0.03 | 0.02 | 0.00 | 0.01 |
|  | Median | 0.23 | 0.46 | 0.27 | 0.19 | 0.01 | 0.12 |
|  | 95 % CI lower - upper | 0.18 - 0.29 | 0.39 - 0.64 | 0.21 - 0.34 | 0.15 - 0.25 | 0.01 - 0.01 | 0.08 - 0.13 |
|  | Min - Max | 0.02 - 0.57 | 0.09 - 1.02 | 0.02 - 0.62 | 0.01 - 0.41 | 0.00 - 0.02 | 0.01 - 0.24 |
|  | Missing | 15 (37.50) | 15 (37.50) | 15 (37.50) | 15 (37.50) | 15 (37.50) | 15 (37.50) |

**S5. ALECSAT: Potency by visit (FAS)**

|  | | % killed at 50*10E3 lymphocytes/well (N=40) | % killed at 100*10E3 lymphocytes/well (N=40) | % killed at 200*10E3 lymphocytes/well (N=40) |
| --- | --- | --- | --- | --- |
|  |  |  |  |  |
|  |  |  |  |  |
| Week8 ALECSAT patients | n | 28 | 28 | 30 |
|  | Mean (SD) | 27.48 (16.57) | 41.42 (22.33) | 56.14 (24.66) |
|  | Standard error of the mean | 3.13 | 4.22 | 4.50 |
|  | Median | 27.30 | 43.67 | 62.95 |
|  | 95 % CI lower - upper | 21.05 - 33.90 | 32.76 - 50.08 | 46.93 - 65.35 |
|  | Min - Max | 2.00 - 59.40 | 4.10 - 73.20 | 6.00 - 86.10 |
|  | Missing | 12 (30.00) | 12 (30.00) | 10 (25.00) |
|  |  |  |  |  |
| Week12 ALECSAT patients | n | 21 | 21 | 24 |
|  | Mean (SD) | 18.38 (14.68) | 29.40 (20.36) | 45.60 (20.98) |
|  | Standard error of the mean | 3.20 | 4.44 | 4.28 |
|  | Median | 13.00 | 24.00 | 44.65 |
|  | 95 % CI lower - upper | 11.69 - 25.06 | 20.14 - 38.67 | 36.74 - 54.46 |
|  | Min - Max | 0.00 - 49.40 | 1.10 - 69.70 | 17.10 - 88.30 |
|  | Missing | 19 (47.50) | 19 (47.50) | 16 (40.00) |
|  |  |  |  |  |
| Week16 ALECSAT patients | n | 26 | 26 | 26 |
|  | Mean (SD) | 22.17 (13.16) | 35.81 (18.45) | 54.37 (22.15) |
|  | Standard error of the mean | 2.58 | 3.62 | 4.34 |
|  | Median | 23.95 | 39.85 | 63.60 |
|  | 95 % CI lower - upper | 16.85 - 27.48 | 28.36 - 43.26 | 45.42 - 63.31 |
|  | Min - Max | 3.40 - 59.80 | 4.90 - 71.00 | 8.60 - 85.10 |
|  | Missing | 14 (35.00) | 14 (35.00) | 14 (35.00) |
|  |  |  |  |  |
| Week34/35AB | n | 17 | 17 | 18 |
|  | Mean (SD) | 24.67 (15.61) | 41.56 (21.47) | 53.90 (25.65) |
|  | Standard error of the mean | 3.79 | 5.21 | 6.05 |
|  | Median | 24.80 | 44.20 | 63.55 |
|  | 95 % CI lower - upper | 16.65 - 32.70 | 30.52 - 52.61 | 41.14 - 66.66 |
|  | Min - Max | 1.00 - 54.20 | 1.80 - 68.90 | 2.00 - 83.00 |
|  | Missing | 23 (57.50) | 23 (57.50) | 22 (55.00) |
