# Supplementary material for: A randomized phase II trial of efficacy and safety of the immunotherapy ALECSAT as an adjunct to radiotherapy and temozolomide for newly diagnosed glioblastoma
Source: Neurooncol Adv. 2021 Oct 22;3(1):vdab156. doi: 10.1093/noajnl/vdab156 (PMC8577524; doi:10.1093/noajnl/vdab156)
Supplement: vdab156_suppl_Supplementary_Data_S1 [file vdab156_suppl_supplementary_data_s1.docx]

Methods Supplement 1

# PROTOCOL SYNOPSIS

| **TITLE:**  An open-label, randomised, Phase II study to investigate the efficacy and safety of ALECSAT treatment as an add-on therapy to radiotherapy and temozolomide in patients with newly diagnosed glioblastoma. |
| --- |
| **PROTOCOL NO:**  CV-006 |
| **INVESTIGATOR STUDY SITES:**  This study will be conducted in four study sites located in Sweden. |
| **OBJECTIVES:**  **Primary Objective:**   - To obtain preliminary but not conclusive evidence by analysing progression-free survival (PFS) in patients receiving autologous lymphoid effector cells specific against tumour cells (ALECSAT) as add-on therapy to radiotherapy and temozolomide (TMZ) (this combination is hereafter entitled standard of care, SOC) versus patients receiving SOC only.   **Secondary Objectives:**   1. To compare overall survival (OS) between patients who received ALECSAT as an add-on therapy to SOC versus patients who received SOC only. 2. To compare OS rate at 12 and 24 months between patients who received ALECSAT as an add-on therapy to SOC versus patients who received SOC only. 3. To assess the safety of ALECSAT treatment. |

| **METHODOLOGY:**  This is a randomised, open-label, multi-centre, Phase II study in patients with newly diagnosed glioblastoma.  Up to 60 patients with newly diagnosed glioblastoma will be enrolled in the study in a 1:2 allocation (SOC vs ALECSAT + SOC).  Patients recruited into this study will receive either:   - ALECSAT as an adjunct therapy to SOC for newly diagnosed glioblastoma (first line therapy: Stupp regimen), followed by second line therapy at the Investigator’s discretion, or - SOC for newly diagnosed glioblastoma (first line therapy: Stupp regimen, followed by second line therapy at the Investigator’s discretion).   Patients will be screened (Week -4 - 0) and will enter the study within six weeks of their glioblastoma resection. Eligible patients will be randomised to either ALECSAT treatment as an add-on therapy to SOC, or SOC only.  Patients will continue to complete the treatment phase. All patients will complete the radiotherapy phase of the Stupp regimen (radiotherapy ideally five days per week combined with daily TMZ for approximately six weeks (Weeks 1-6). Patients who terminate radiotherapy or TMZ treatment early for any reason will be allowed to remain in the study.  Patients will begin adjuvant TMZ treatment four to five weeks after completion of radiotherapy. Typically, six cycles of TMZ will be given (daily for five days every 28 days).  Alongside TMZ treatment, patients randomised to the ALECSAT treatment arm will receive three doses of ALECSAT at four week intervals during the loading phase of the study (Weeks 8-16). Following the loading phase, patients will enter the maintenance phase. Patients in the ALECSAT treatment arm will receive further ALECSAT administrations during the maintenance phase at 12 week intervals except for the first maintenance dose, which is given after approximately 16 weeks have elapsed since the loading phase. The maintenance phase of ALECSAT administrations will continue for patients in the ALECSAT treatment arm until death or until a patient discontinuation criterion is observed or until closure of the study (24 months after recruitment has closed). After termination of the study, patients treated with ALECSAT will be offered to continue ALECSAT treatment in a compassionate use program.  Patients randomised to the control arm (Stupp treatment only followed by second line treatment at the discretion of the Investigator) will follow the same study plan and undergo the same study procedures as patients in the ALECSAT treatment arm (with the exception of blood donations for ALECSAT production, ALECSAT administrations and vital signs). After termination of the study, patients treated in the SOC arm will continue treatment as judged by the investigator. |
| --- |

| The study discontinuation criteria are as follows:   - Withdrawal of consent - An adverse event which requires discontinuation of the study medication or results in inability to continue to comply with study procedures - Disease progression which results in inability to continue to comply with study procedures - Major protocol deviation - The blood donation for ALECSAT production may put the patient at risk - Exclusion criteria met.   Patients randomised to the ALECSAT treatment arm will be allowed to remain on ALECSAT treatment if disease progression is observed and second line treatment is initiated.  The final analysis will be carried out when 47 events (investigator assessed progression or death by any cause) have been observed. Twenty-four months after recruitment to the study has closed, treatment and data collection for any patients still alive will end and the study will close. Any data collected for patients still alive after the point of final analysis (47 events) until study closure (24 months after recruitment has closed) will be analysed and included as an addendum to the final report.  There will be an independent Data Safety Monitoring Board (DSMB) performing annual review of all collected safety data. |
| --- |
| **NUMBER OF PATIENTS:**  Up to 60 patients will be enrolled. |
| **INCLUSION/EXCLUSION CRITERIA:**  **Inclusion:**   1. Male or female patients, aged between 18 and 70. 2. Histologically confirmed, newly diagnosed glioblastoma, including gliosarcoma. 3. Eligible for combined radiotherapy and TMZ treatment (Stupp treatment). 4. Patients with complete or partial tumour resection. For patients with limited tumour volume, biopsy is acceptable. 5. WHO Performance status 0-2. 6. Body weight ≥ 40 kg (males), ≥ 50 kg (females). 7. Able and willing to provide written informed consent and comply with the study protocol and study procedures. 8. Women of child-bearing potential must have a negative pregnancy test at screening |

| and agree to use acceptable methods of contraception during the study.  **Exclusion:**   1. Prior treatment for brain tumours at study entry. 2. Prior treatment with temozolomide at study entry 3. Females who are pregnant, planning to become pregnant or breastfeeding 4. Positive tests for anti- human immunodeficiency virus (HIV)-1/2; HBsAg, anti-HBc, anti-HCV or being positive in a Treponema Pallidum test (syphilis). 5. Patients who may have been exposed to high risk contagious virus within a reasonable time prior to enrolment should be excluded, unless the patient has been tested negative.   e.g. by travelling in areas of the world with known high risk of infection or known epidemics, (in particular but not limited to West Nile virus (in season), Dengue fever, Zika or Ebola when outbreaks are recognized)   1. Patients from high incidence areas for Human T-Lymphotropic Virus (HTLV-1) virus or who has a parent or spouse from a high incidence area must be excluded unless tested negative for HTLV-1 virus. 2. Known allergy to study medication. 3. Any condition or illness that, in the opinion of the Investigator or medical monitor, would compromise patient safety or interfere with the evaluation of the safety of the investigational drug. 4. Any concurrent illness that may worsen or cause complications in connection with blood donation, for example uncontrolled epilepsy, cardiovascular, cerebrovascular or respiratory disease. 5. Use of immunosuppressant drugs with the exception of steroids. 6. Blood transfusion within 48 hours prior to the donation of blood for ALECSAT production. 7. Low haemoglobin count in the opinion of the Investigator. 8. Lymphocyte count <0.3 x 10^9^/litre. 9. Receiving any other experimental treatment, including compassionate use programs and other interventional clinical studies for glioblastoma, within 30 days prior to inclusion, at the moment of inclusion or during active treatment within the assigned group. 10. TMZ contraindication. |
| --- |
| **DOSE/ROUTE/REGIMEN:**  **For patients randomised to ALECSAT treatment only**:  Product name: ALECSAT  Dose: 1 x 10^7^- 1 x 10^9^ cells suspended in 20 ml plasmalyte solution  Dosing schedule: Loading phase (Weeks 8-16) - approximately three doses administered at approximately four week intervals. Maintenance phase (from Week 18) - dose administered |

| 12 week intervals except for the first maintenance dose which is given after approximately 16 weeks have elapsed since the loading phase until death or until a patient discontinuation criterion is observed or until closure of the study (24 months after last patient first visit (LPFV)).  Pharmaceutical form: Suspension for injection Route of administration: Intravenous  Patients randomised to ALECSAT treatment will also receive the SOC treatment as stated below. |
| --- |
| **SOC TREATMENT:**  The SOC therapy for newly diagnosed glioblastoma is the Stupp regimen. The Stupp treatment includes a combination of external radiotherapy (daily fractions of 2 Gy per fraction ideally five days per week up to a total dose of 60 Gy) and TMZ (75 mg/m^2^) daily for approximately six weeks. Patients who terminate radiotherapy or TMZ treatment early for any reason will be allowed to remain in the study.  Patients then begin adjuvant TMZ treatment four to five weeks after completion of radiotherapy. Typically, six cycles of TMZ will be given (daily for five days every 28 days). TMZ will be administered orally at a dose of 150 mg/m^2^ per day for the first treatment cycle. The dose of TMZ will increase to 200 mg/m^2^ per day in the subsequent treatment cycles, in the absence of haematological toxicity. No other first line treatment is allowed.  Patients may also receive any second line/salvage therapy, at the discretion of the Investigator as per institutional routine. |
| **CRITERIA FOR EVALUATION:**  ***Efficacy:***  Primary Endpoint:   - PFS is measured as the time from surgical resection to the date of investigator assessed progressive disease (PD) or death by any cause. PD will be declared based on MRI scans, clinical status, and corticosteroid usage according to the Revised Assessment for Neuro-Oncology (RANO) criteria.   Secondary Endpoints:   1. OS time is measured from time of surgical resection until death for any reason. 2. Proportion of patients alive at 12 and 24 months after randomization (1-year and 2- year OS).   Safety Endpoints:   - - Adverse events and pregnancies   - Safety laboratory parameters |

| - Immune system status   Exploratory Endpoint:   - PFS measured as the time from surgical resection to the date of PD centrally assessed |
| --- |
| STATISTICAL METHODS:  All data will be presented in patient data listings. Data will be summarised by treatment groups. For continuous variables, descriptive statistics (n, mean, median, standard deviation, minimum, and maximum) will be presented. For categorical variables, frequencies and percentages will be presented. Graphical displays will be presented as appropriate.  *Power and Sample Size:*  This is a phase II screening design (Rubinstein et al. 2005) providing preliminary but not conclusive evidence of efficacy.  The assumptions of the sample size calculation are:   - The Type I error rate (alpha) is set to 10% for a one-sided test; - The Type II error rate should be not more than 20% (Power= 80%); - The median PFS of patients treated with SOC is assumed to be 6.9 months [[1](#_bookmark127)]   The expected hazard ratio in favour of the ALECSAT+ SOC therapy is assumed to be 0.54, which is equivalent to a median PFS of 12.8 months on ALECSAT+SOC assuming an exponential survival distribution. Under these assumptions, the number of investigator assessed events (progressions) required for the final analysis is 47. A total of 60 patients will be randomised to the study in a 1:2 allocation (SOC: ALECSAT as an adjunct therapy to the SOC).  *Interim Analyses*  No interim analyses are planned.  *Analysis of data*  Analysis of PFS, based on investigator-declared PD or death, will take place when 47 events (PD or death) have been registered. Any data collected after this time point up to study closure (24 months after LPFV) will be analysed and included as an addendum to the final report.  *Demographics and Baseline Characteristics:*  Demographic data, like age and gender, will be described using n, mean, median, standard deviation, minimum, and maximum for age and percentages for gender.  Baseline characteristics with respect to the disease:   - First symptoms - Local Tumour (Frontal / Temporal / Parietal / Occipital / Central /Bilateral Multifocal) - WHO Performance status at time of enrolment (0, 1, 2) |

| - Isocitrate Dehydrogenase 1 (IDH1) status - O^6^-Methylguanine-DNA-Methyltransferase (MGMT) status - Extent of resection (complete, partial or biopsy) - Systemic steroid treatment will be described by frequency tables.   *Efficacy:*  The primary endpoint, PFS, will be analysed using the Kaplan-Meier survival estimate and compared between the treatment groups using a log-rank test. OS will be analysed in the same manner. Estimates of median OS and PFS will be calculated using the Kaplan-Meier method. 95% confidence intervals for the median survival times will be presented.  The proportion of subjects alive at 12 and 24 months will be analysed by treatment group using chi-square tests and logistic regression. The logistic regression model will include the same covariates as described above for the Cox Proportional hazards model.  *Safety:*  Adverse events will be displayed using frequency table methods, presenting number of patients and percentages.  Safety laboratory parameters will be presented as mean values, standard deviations, medians and minimum and maximum values. |
| --- |
