# Supplementary material for: A randomized phase II trial of efficacy and safety of the immunotherapy ALECSAT as an adjunct to radiotherapy and temozolomide for newly diagnosed glioblastoma
Source: Neurooncol Adv. 2021 Oct 22;3(1):vdab156. doi: 10.1093/noajnl/vdab156 (PMC8577524; doi:10.1093/noajnl/vdab156)
Supplement: vdab156_suppl_Supplementary_Data_S2 [file vdab156_suppl_supplementary_data_s2.docx]

Methods Supplement 2

**Production of ALECSAT**

The ALECSAT production was performed in GMP facilities at Cytovac A/S. The process is initiated by a patient’s donation of peripheral blood at the local hospital, and subsequent isolation of the monocytes and lymphocytes as described previously (Kirkin et al, 2018). The manufacturing process for ALECSAT takes 26 days and includes 4 overall steps: 1) preparation of the mature dendritic cells, 2) preparation of proliferating culture of the CD4+ (T_H_) cells, 3) induction of the expression of cancer/testis antigens by treatment of activated lymphocytes with 5-aza-2’deoxycytidine, a DNA-demethylating agent, 4) stimulation of non-activated lymphocytes by the cancer/testis antigen-expressing activated T_H_ cells and their final short-term expansion.

**Measurement of MAGE antigen expression by Quantitative Real time PCR Analysis**

Total RNA was isolated from lymphocytes using the NucleoSpin RNA Plus kit (Macherey-Nagel), and reverse transcribed using SuperScript™ IV VILO™ Master Mix with ezDNase™ Enzyme (Invitrogen) and random hexamers and oligo (dT24) primers. cDNA was amplified in a LightCycler Nano (Roche) using the FastStart Essential DNA Green Master mix (Roche) and previously described primers and conditions (Weinert et al, 2009), except primers for MAGE-A4, provided by RealTimePrimers.com. The data were normalized to GAPDH expression.

**Table A.** Primer sequences for Q-PCR measurement of MAGE gene expression.

| **Gene** | **Primer sequence (5´-3´)** |
| --- | --- |
| MAGE A1, q s | AGTAGTAGGTTTCTGTTCTATTGGG |
| MAGE A1, q a | TACTTATTCCACTGCTGTTATTATCC |
| MAGE A3, q s | GCTGAGTGTGTTAGAGGTGTT |
| MAGE A3, q a | AGGGGTGGGTAGGAAATGT |
| MAGE A4, q s | TGTGATCTTCGGCAAAGCCT |
| MAGE A4, q a | TTTCCTGCACCCAATCTTGG |
| MAGE A6, q s | GCTGAGTGTGTTAGAGGTGTT |
| MAGE A6, q a | CAGGAGTGGGTAGGAAATGC |
| MAGE A10, q s | CCTGCCAGACAGTGAGTCTT |
| MAGE A10, q a | TGGGATCCACCTCCTTTACA |
| MAGE A12, q s | CTGAGTGTGTTGGAGGCATC |
| MAGE A12, q a | GGTGGGTAGGAAATGTGAGGT |
| GAPDH, q s | AGCTTGTCATCAATGGAAATCCC |
| GAPDH, q a | GTGAAGACGCCAGTGGACTC |

***In vitro* testing of lytic activity of ALECSAT**

The lytic activity of the ALECSAT product was tested against glioblastoma cell line HROG17 (Mullins CS et al., 2013) using real time cytotoxicity assay and the RTCA iCELLigence system (ACEA Bioscience, San Diego, USA). Tumor cells were seeded at density 3x10^4^ cells per well in a total volume of 400 µL of RPMI 1640 medium with 10% FCS. After 3 or 20 hours, different doses of ALECSAT-lymphocytes, (50x10^3^, 100x10^3^ and 200x10^3^ respectively), were added in 200 µL of AIM-V medium. Killing of tumor cells was associated with decrease in cell impedance (measured as dimensionless Cell Index). Cell Index was normalized to the time point of addition of the effector lymphocytes. Total reaction time was either 6 hours, or 20-24 hours. Cytotoxic activity (percentage) was calculated as 100 – Normalized Cell Index. This test mainly measured the lytic activity of NK cells present in ALECSAT preparations, and to a lesser extent NK-like non-specific activity of CTLs.

**Flow cytometry**

For determination of surface expression of different markers on the cells, the directly conjugated antibodies were used, according to Kirkin et al. 2018. The recommended isotypic controls were used for the phenotyping of the cells. The cell samples were analyzed using FC500 MPL Flow Cytometer and the CXP analytical software (Beckman Coulter).

**Table B**. Antibodies used for FACS

| **Target** | **Conjugation** | **Catalog No.** | **Supplier** |
| --- | --- | --- | --- |
| CD3 | FITC | IM1281 | Beckman |
| CD4 | FITC | 555346 | BD Biosciences |
| CD8 | PE | 555635 | BD Biosciences |
| CD27 | PE | 555441 | BD Biosciences |
| CD56 | PE | 555516 | BD Biosciences |
| CD62L | PE-Cy5 | IM2655 | Beckman |
| CD80 | FITC | 557226 | BD Biosciences |
| CD83 | PE | 556855 | BD Biosciences |
| CD86 | PE | 555658 | BD Biosciences |

References (Supplementary Methods):

Kirkin AF et al. Adoptive cancer immunotherapy using DNA-demethylated T helper cells as antigen-presenting cells. Nat Commun. 2018;9(1):785.

Weinert BT et al. Real-time PCR analysis of genes encoding tumor antigens in esophageal tumors and a cancer vaccine. Cancer Immun. 2009;9:9.

Mullins CS et al. A Comprehensive Approach to Patient-individual Glioblastoma Multiforme Model Establishment. Journal of Cancer Science & Therapy. 2013;05(12).
